# Secretary West and the Buffet Banquet

**Published:** 1893-08

**Authors:** D. R. Wallace

**Affiliations:** Waco


### Correspondence.

For Texas Medical Journal.

### SECRETARY WEST AND THE BUFFET BANQUET.

Vixerē fortes ante Agamemnona Multi.—HORACE. *

MESSRS. EDITORS:—In your June issue H. A. West, M. D., in an article, "Notes on the Recent Meeting in Galveston," writes:

"Of the social features of the occasion it would not be becoming in the writer to speak in detail, but one thing I wish to mention: that is, what appears to me as the charming success of substituting the buffet banquet and concert, where ladies could grace the occasion by their presence and smiles, for the traditional drinking bout and maudlin speech making, ordinarly called a banquet, which has hertofore prevailed. It was mainly to try this experiment that induced me to accept the responsibility and labor incident to the chairmanship of the Committee of Arrangement. The general impression made upon my mind was that the recent meeting was a grand success in every way, and a most favorable augury for the future progress and usefulness of our beloved old Assocaition."

These words are quite in keeping and accord with his speech of acceptance when elected Secretary of the Association. Little credit was then accorded the body, and less to the former secretaries, the distinguished Young, the laborious East, the painstaking Burt, the brilliant Bibb, the versatile Daniel. He was "induced" (pro bono publico) "to take the place to elevate the tone, and increase the scientific character of the Association." Two meetings have been held since his election, and the two meetings previous to his advent (the one at Fort Worth, the other at Waco), suffer nothing in comparison. Nobody has been startled that I have heard of, by any brilliant *coup d' etat* of the new Secretary.

---

[* There lived many heroes before Agamemnon.—ED.]

The zealous doctor now a second time (pro bono publico, of course,) descends into the arena as a reformer in the character of Chairman of the Committee of Arrangements, in which role (himself the judge) he scored a "charming success,"—"substituting a buffet banquet and concert where ladies could grace the occasion with their presence and smiles for the traditional drinking bout and maudlin speech making."

Now just what this meteoric display of buffet oratory means, I am sure I do not quite comprehend. I have known the Texas State Medical Association from its first meeting. I have never witnessed, never heard of any drinking bouts or maudlin speech-making, much less that they were traditional. *He* substituted—*he* tried the experiment. There were banquets with wine, and banquets without wine, long before this great reformer and experimenter became a member. I have been a member from the first meeting and I have seen but one drunken man at a banquet of the Association,—he made a maudlin speech. He was not a member, was not a medical man. At the request of the Chairman of Committee of Arrangements of the banquet at Fort Worth (1890), the last I attended where wine flowed plentifully, I presided. I thought I saw what passed. I saw no drinking bout, heard no maudlin speech-making. I did hear some fine speeches, well suited to a convivial occasion, speeches that added no little to the merriment of the guests, and helped to make the banquet a "charming success." I saw too quite a number of beautiful ladies of high social standing gracing the occasion by their presence and smiles.

The doctor assures us he made his banquet a charming success. Admit *he* did it all—that the other members of the committee were dead-heads—did nothing. Be his whatever merit he may claim, it is no concern of mine. But I must, I will protest, with whatever emphasis I may, against the unjust, uncalled for, gratuitous insult to gentlemen, his peers in every respect, who were just as praiseworthy and successful in their efforts to entertain the Association on previous occasions as was Dr. West at Galveston,—and this for no other or better reason than that these gentlemen differed with him in regard to the propriety of using wine upon such an occasion.

Three or four decades ago, when the writer first came to the State, it was a custom when a newcomer was observed to begin to swell with self-importance, and to talk about how they did things back where he came from, and to put on airs, to tip him a gentle reminder that it was the thing for him to do to *go foot and spell up*. I do not forget that customs are like laws—

"Laws, as we read in ancient sages,
Have been like cobwebs in all ages.
Cobwebs for little flies are spread,
And laws for little folks are made;
But if an insect of renown
Hornet or beetle, wasp or drone,
Be caught in quest of sport or plunder
The flimsy fetter flies in sunder."

The paragraph quoted from Dr. West's letter is the more to be regretted, as it is not calculated to help the T. S. M. A. in which all desire harmony; as little is it of a character to conciliate the profession and draw them to the support of the Medical Department of the Texas State University with which Dr. West is connected, and for the up building of which the noble and learned Doctors Paine and Clopton and others are laboring so indefatigably, and for the success of which all Texas medical men who care for the status of scientific rational medicine in their State should heartily co-operate. D. R. WALLACE.

